# Lactic acid Bacteria isolated from European badgers (*Meles meles*) reduce the viability and survival of Bacillus Calmette-Guerin (BCG) vaccine and influence the immune response to BCG in a human macrophage model

**DOI:** 10.1186/s12866-018-1210-z

**Published:** 2018-07-13

**Authors:** Anna Stedman, Carlos Maluquer de Motes, Sandrine Lesellier, Deanna Dalley, Mark Chambers, Jorge Gutierrez-Merino

**Affiliations:** 1 School of Biosciences and Medicine, University of Surrey, Guildford, GU2 7XH UK; 2 The Pirbright Institute, Woking, GU24 0NF UK; 3 Bacteriology Department, Animal and Plant Health Agency, Addlestone, KT15 3NB UK; 4 School of Veterinary Medicine, University of Surrey, Guildford, GU2 7AL UK

**Keywords:** Lactic acid bacteria, Bovine tuberculosis, BCG, Oral vaccination, Badgers

## Abstract

**Background:**

Bovine tuberculosis (bTB) caused by *Mycobacterium bovis* is the most serious endemic disease affecting livestock in the UK. The European badger (*Meles meles*) is the most important wildlife reservoir of bTB transmission to cattle, making eradication particularly difficult. In this respect, oral vaccination with the attenuated *M. bovis* vaccine Bacillus Calmette-Guerin (BCG) has been suggested as a wide-scale intervention to reduce bTB infection in badgers. However, experimental studies show variable protection. Among the possibilities for this variation is that the resident gut bacteria may influence the success of oral vaccination in badgers; either through competitive exclusion and/or inhibition, or via effects on the host immune system. In order to explore this possibility, we have tested whether typical gut commensals such as Lactic Acid Bacteria (LAB) have the capacity to impact on the viability and survival rate of BCG and to modulate the immune response to BCG using an in vitro model.

**Results:**

Twelve LAB isolated from badger faeces displayed inhibitory activity to BCG that was species-dependent. Weissella had a bacteriostatic effect, whereas isolates of enterococci, lactobacilli and pediococci had a more bactericidal activity. Furthermore, BCG-induced activation of the pro-inflammatory transcription factor NF-κB in human THP-1 macrophages was modulated by LAB in a strain-dependent manner. Most pediococci enhanced NF-κB activation but one strain had the opposite effect. Interestingly, isolates of enterococci, lactobacilli and weissella had different effects as immunomodulators of BCG-induced macrophage responses as some had no significant influence on NF-κB activation, but others increased it significantly.

**Conclusions:**

Our in vitro results show that LAB isolated from badgers exhibit significant inhibitory activity against BCG and influence the immune activation mediated by BCG in a human macrophage assay. These findings suggest that gut commensal bacteria could play a role in influencing the outcome of oral BCG vaccination. Inactivated cells of LAB, or LAB that are bacteriostatic but have a synergistic immunostimulatory effect with BCG, could be potential adjuvants to be used for oral vaccination in badgers. Further work is needed to take into account the complex nature of the gut microbiome, specific immunity of the badger and the in vivo context.

**Electronic supplementary material:**

The online version of this article (10.1186/s12866-018-1210-z) contains supplementary material, which is available to authorized users.

## Background

Bovine tuberculosis (bTB) caused by *Mycobacterium bovis* is the most serious endemic disease affecting livestock in England and Wales [1]. Scientific evidence suggests that the European badger (*Meles meles*) is one of the most significant wildlife reservoirs of *M. bovis* in parts of the UK and Ireland [2]. The cases of bTB infection across England and Wales appear to occur more frequently in regions with higher densities of badgers [3], which have led both governments to implement comprehensive control strategies, including tighter cattle movement controls, biosecurity measures, culling and vaccination of badgers [4].

Biosecurity measures intended to keep badgers and cattle apart can be hard to implement at pasture [5]. In this respect, vaccination with the attenuated *M. bovis* vaccine Bacillus Calmette-Guerin (BCG) could be a viable alternative to control *M. bovis* infection in badgers [6]. However, the use of injectable BCG is limited and expensive due to the need to trap animals in order to vaccinate them [7, 8]. Oral BCG vaccination could bring the prospect of a wider-scale implementation and has been reported to reduce not only bTB infection in badgers but also their susceptibility to infection [9, 10]. However, protection with oral BCG is variable, at least in experimental models [11]. Although the reasons for this variability in protection have not been accounted for conclusively, one possibility could be the influence of gut resident bacteria on the efficacy of orally administered BCG due to competitive exclusion and/or inhibition in the gut; or after uptake by the oral mucosa; or through direct and indirect effects on immune cells [12] .

The composition of the mammalian intestinal microflora varies considerably over time and between individuals [13], and badgers are likely to be no different. Therefore, we sought to focus on Lactic Acid Bacteria (LAB) for the purposes of our initial investigations. LAB are gut commensal bacteria that provide a beneficial effect on intestinal epithelial cells in numerous ways: they metabolize indigestible compounds; provide the host with essential nutrients; contribute to the development of the intestinal architecture; and prevent against colonization of opportunistic pathogens [14]. It is very well documented that LAB produce antimicrobial compounds such as organic acids, hydrogen peroxide, ethanol and bacteriocins [15], which can be active against a broad range of bacteria including mycobacteria [16, 17]. Furthermore, LAB are key to maintaining the intestinal microbial balance in mammals, as well as to generate beneficial host immunomodulation [13]. The gut immune system recognizes LAB via the ligation of microbe-associated molecular patterns (MAMPs) to pattern recognition receptors (PRRs) such as Toll-like receptors (TLRs) that are expressed on immune cells and epithelial cells [18]. Upon MAMP recognition, PRRs activate an intracellular signalling cascade converging on the pro-inflammatory transcription factor NF-κB, which is crucial in maintaining immune homeostasis and regulating immunological and anti-microbial responses in barrier tissues such as the intestine [19]. Therefore, LAB play a role in innate immune responses and modulate responses to oral vaccination [20].

The aim of this study was to isolate LAB naturally excreted by badgers in order to identify those isolates that display inhibitory activity to BCG and assess their ability to modulate BCG-mediated pro-inflammatory responses in an established surrogate macrophage model. LAB isolated from badger faeces were initially screened for inhibitory activity against non-pathogenic *Mycobacterium smegmatis*, with a total of 40 LAB showing this activity. From these isolates, 12 were selected to be most representative and co-cultured with BCG to evaluate their antagonistic influence on the viability and survival rate of BCG. To study their potential as immunodulators, the human macrophage line THP-1 was exposed to the 12 LAB strains in isolation and in the presence of BCG, and the inflammatory responses recorded in the form of NF-κB activation and TNF-α production. Our results demonstrate that numerous LAB isolated from badgers have the capacity to reduce the viability and survival of BCG in vitro and to modulate the immune response of human THP-1 macrophages exposed to BCG. These fundamental findings suggest that gut commensal bacteria could play a role in influencing the outcome of oral BCG vaccination in badgers. In this respect, in vivo work will be needed to further explore this possibility.

## Results

### Isolation of LAB from badgers with antimycobacterial activity

**Fig. 1:**
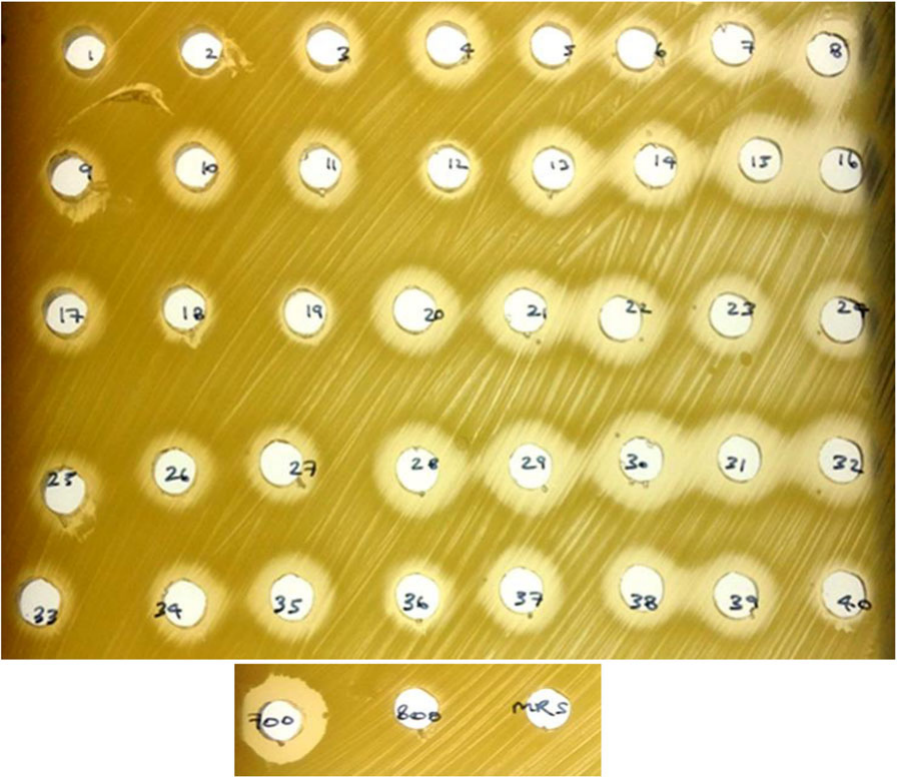
Agar-well Diffusion Test showing the antimicrobial activity of cell-free supernatants collected from cultures of 40 LAB isolated from badgers against M. smegmatis. These 40 isolates were identified by 16S rRNA sequencing and named as: (1) *Pediococcus acidilactici* A5; (2)* P. lolii* A6; (3) *Enterococcus faecalis* A7; (4) *Weissella cibaria* A23; (5) *W. paramesenteroides* A37; (6) *P. pentosaceus* B4; (7) *P. acidilactici *B41; (8) *P. lolii *B53; (9) *E. faecalis* C34; (10) *Lactobacillus reuterii* D4; (11) *P. acidilactici* E12; (12) *P. lolii *E23; (13) *P. acidilactici *E24; (14) *P. acidilactici* E48; (15) *P. lolii* F7; (16) *P. acidilactici* F20; (17) *P. acidilactici* F24; (18) *P. acidilactici *F44; (19) *E. faecalis* F48; (20) *P. lolii* G23; (21) *P. pentosaceus* G24; (22) *P. acidilactici* G41; (23) *E. faecalis* G42; (24) *P. lolii *G54; (25)* P. acidilactici* H33; (26)* E. faecalis* H34; (27) *P. acidilactici *I32; (28) *E. faecium* I47; (29) *L. plantarum* J2; (30) *P. acidilactici *J26; (31) *P. acidilactici* L4; (32) *P. acidilactici* M16; (33) *P. acidilactici* M17; (34) *W. paramesenteroides* N43; (35) *E. faecium* O44; (36) *L. plantarum* P5; (37) *P. acidilactici* Q16; (38) *L. plantarum* R20; (39)* L. plantarum* S48; (40) *L. plantarum* T17. All supernatants showed very acidic pH ranging between 3.6–-4.0 for lactobacilli and pediococci, and 4.2–-4.5 for weissella and enterococci. MRS broth (MRS) and supernatants from cultures of *L. lactis* NZ9700 (700) and *L. lactis* NZ9800 (800) were used as controls. Both L. lactis strains reduce pH in MRS but no lower than pH 4.5

A total of 40 LAB isolated from badger faeces showed antimicrobial activity against *Mycobacterium smegmatis* and were identified as enterococci, lactobacilli, pediococci and weissella (Figure 1). At this stage *M. smegmatis* was used as a fast-growing mycobacteria species to facilitate rapid antimycobacterial screening [21]. As indicated in Table 1, these 40 isolates with antimicrobial activity against *M. smegmatis* were randomly distributed between sexes and the weight of the badgers and/or the absolute or relative total counts observed on selective agar plates for LAB (De Man, Rogosa, Sharpe, MRS) and total viable counts (Plate Count Agar, PCA).

**Table 1:** Total counts of microaerophilic bacteria and bacterial isolates displaying antimycobacterial activity from faecal samples of the 26 badgers selected for this study

Badgers:			Total Counts:			Isolates with antimycobacterial activity^f^
IC^a^	Sex^b^	Weight (Kg)	MRS^c^	PCA^d^	MRS:PCA^e^	
A	f	8.3	5.61	5.56	1.14	5, 6, 7, 8, 23, 37
B	m	10.1	5.98	5.93	1.12	4, 41, 53
C	f	8.2	6.36	6.38	0.96	34
D	m	10.2	7.54	7.61	0.85	4
E	f	7.5	5.72	5.71	1.02	12, 23, 24, 48
F	m	12.1	7.32	6.34	9.54	7, 20, 24, 44, 48
G	f	9.1	7.46	6.45	10.35	23, 24, 41, 42
H	f	8.8	6.70	6.40	2.00	33, 34
I	f	11.5	6.08	6.26	0.67	32, 47
J	f	10.7	5.38	5.40	0.96	2, 26
K	f	10.1	7.92	7.76	1.43	–
L	f	8.8	8.86	8.87	0.97	4
M	m	12.3	8.45	8.43	1.04	16, 17
N	m	8.4	8.95	8.08	0.75	43
O	f	8.0	8.76	8.87	0.78	44
P	m	10.8	8.38	8.53	0.71	5
Q	f	8.4	8.72	8.87	0.72	16
R	f	8.8	8.13	8.15	0.96	20
S	m	9.9	7.66	7.65	1.02	48
T	f	10	7.41	7.32	1.24	17
U	m	10.5	8.23	8.40	0.68	–
V	m	9	8.48	8.51	0.94	–
W	f	10	7.46	6.86	4.03	–
X	m	10	7.96	6.94	10.46	–
Y	m	12.4	7.08	6.56	3.33	–
Z	m	9.9	7.94	7.49	2.8	–
AA	m	9.1	7.93	6.92	10.24	–
AB	f	8.8	7.72	6.04	4.82	–

^a^Identification code

^b^Female (f) or Male (m)

^c^Total counts on MRS agar plates expressed as log_10_

^d^Total counts on PCA plates expressed as log_10_

^e^Total Counts ratio between MRS and PCA plates

^f^Isolates from MRS that display antimicrobial activity against *M. smegmatis* on a stab-on-agar test. These isolates were named with the numbers indicated in the column and preceded with a letter from their corresponding animal IC

### Viability of BCG in combination with LAB

**Fig. 2:**
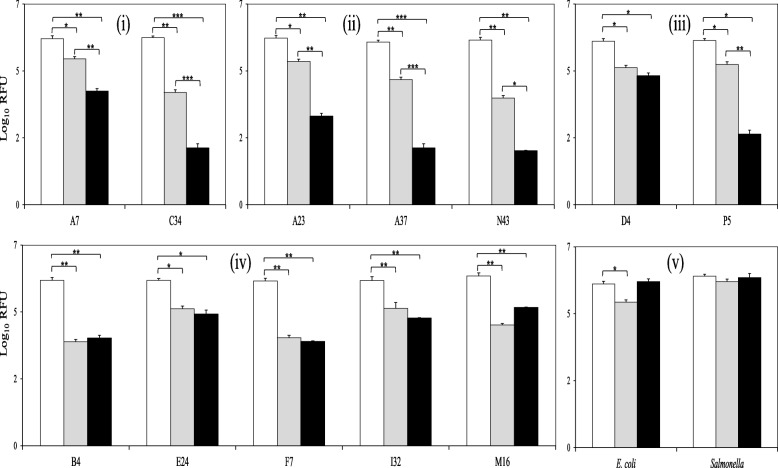
BCG viability when combined with badger isolates of enterococci (i), weissella (ii), lactobacilli (iii) and pediococci (iv) after 0 (white bars), 24 (grey bars) and 48 (black bars) h of incubation. Co-cultures of BCG with gut-associated, Gram-negative bacteria (v) were also monitored for comparative purposes. BCG viability is expressed as log10 Relative Fluorescence Units (RFU). Data are mean ± SD with statistical analysis (Student’s t-test, *p < 0.05, **p < 0.01, ***p < 0.005) and are representative of 3 biological replicates each

Twelve out the 40 LAB isolates with antimycobacterial activity were selected to test their influence on the fitness of BCG (referred as BCG viability in this study). These twelve LAB isolates were: 1, *Enterococcus faecalis* A7; 2, *Weissella cibaria* A23; 3, *W. paramesenteroides* A37; 4, *Pediococcus pentosaceus* B4; 5, *E. faecalis* C34; 6, *Lactobacillus reuterii* D4; 7, *P. acidilactici* E24; 8, *P. lolii* F7; 9, *P. acidilactici* I32; 10, *P. acidilactici* M17; 11, *W. paramesenteroides* N43; and 12, *L. plantarum* P5; which represented four different genera – *Enterococcus*, *Weissella*, *Pediococcus* and *Lactobacillus* - with two strains of the same genus and/or species from at least two different animals, as well as two or more different strains within the most predominant genus and/or species. The 12 selected isolates were then cultured with a BCG strain carrying a Green Fluorescence Protein (GFP) expression vector in order to monitor the fluorescence emission for 48 h (h), as a marker of cell viability. *E. coli* and *Salmonella* were also co-cultured with BCG to provide results with other typical fast-growing enteric bacteria. As illustrated in Figure 2., all LAB isolates reduced BCG viability significantly after 24 and 48 h, especially isolates of enterococci and weissella. No significant differences were observed in the *E. coli* co-cultures after 48 h, nor with *Salmonella* at either time point (24 or 48 h), demonstrating that the activity was specific to LAB and not due to trivial factors, such as competition for nutrients with other faster-growing bacteria.

### Survival of BCG in combination with LAB

**Fig. 3:**
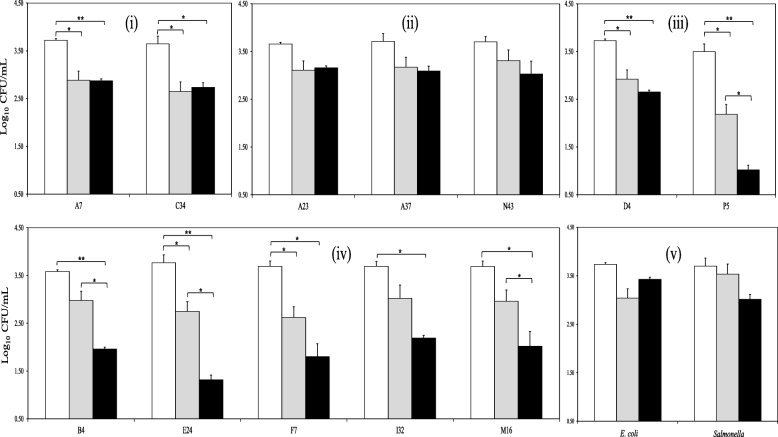
BCG survival rate when combined with badger isolates of enterococci (i), weissella (ii), lactobacilli (iii) and pediococci (iv) after 0 (white bars), 24 (grey bars) and 48 (black bars) h of incubation. Co-cultures of BCG with gut-associated, Gram-negative bacteria (v) were also monitored for comparative purposes. BCG survival is expressed as log10 CFU/mL. Data are mean ± SD with statistical analysis (Student’s t-test, *p < 0.05, **p < 0.01, ***p < 0.005) and are representative of three biological replicates each

The survival of BCG when co-cultured with LAB, *E. coli* or *Salmonella*, was determined by total bacterial counts. With the exception of the weissella isolates, all the LAB combinations resulted in a significant reduction in BCG survival (Figure 3); being particularly evident in the case of the lactobacilli isolates and most of the pediococci. Neither the weissella, *E. coli* nor *Salmonella* had a significant effect on the BCG counts. Taken together with the results for GFP expression, we have shown that isolates of enterococci, lactobacilli and pediococci reduce significantly the viability and survival of BCG, whereas co-cultures with weissella or enterobacteria do not. We next sought potential explanations for these results; whether altered pH or metabolites from LAB could account for the results we observed.

### Effect of pH on BCG with or without LAB

The pH of the co-cultures described above was monitored over 48 h. A significant decrease in pH was recorded in all co-cultures after 24 and 48 h but no significant differences were observed between cultures, including LAB, *E. coli* and *Salmonella* (Additional file 1: Figure S1). The only exception was isolate P5 after 24 h for reasons that remain unknown. Furthermore, both the viability (GFP expression) and survival rate (bacterial counts) of BCG as a monoculture showed no significant changes at pH 7 or pH 5 after 24 and 48 h (Additional file 1: Figure S2). With the exception of isolate P5, all these results confirm that low pH may be insufficient on its own to cause a detrimental effect on BCG.

### Effect of metabolites from LAB on the viability and survival rate of BCG

In order to test whether the antimicrobial effects observed against BCG in the presence of LAB was due to an accumulation of toxic metabolites from LAB, we monitored the viability and survival rate of BCG in cell-free supernatants obtained from mono-cultures of LAB after 48 h of incubation. Supernatants taken from 48 h cultures of *E. coli* and *Salmonella* were also used as controls. No significant reductions were observed with any of the culture supernatants, either in terms of viability or as survival rate (Additional file 1: Figure S3 and S4, respectively). This demonstrated that the negative effect of LAB on BCG was only observed when the bacteria were present together, rather than through the indirect accumulation of antimicrobial metabolites derived from the LAB, such as organic acids (e.g. lactic acid).

### NF-κB response in macrophages exposed to LAB

**Fig. 4:**
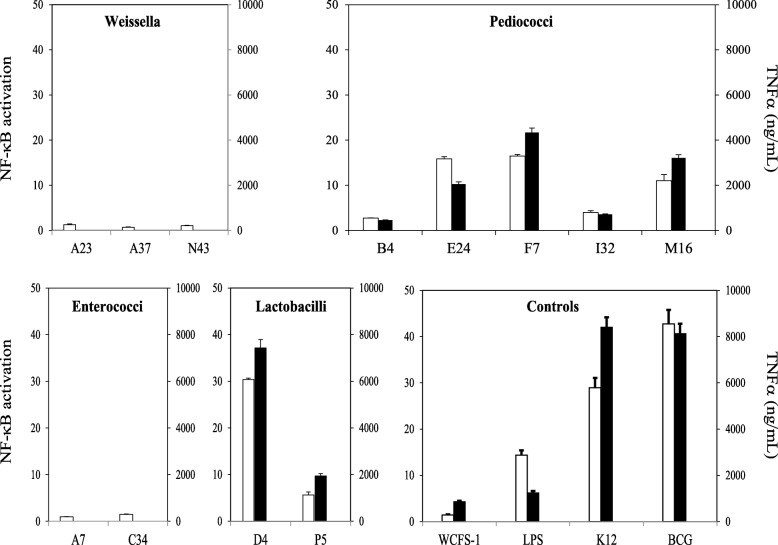
NF-κB activation (white bars) and TNF-α production (black bars) in PMA-differentiated THP-1 macrophages exposed to inactivated cells from LAB isolates of weissella, pediococci, enterococci and lactobacilli at a ratio of 1 macrophage per 100 bacteria for 24 h. NF-κB activation is presented as a fold increase over a non-stimulated condition on the primary Y axis (left hand), while TNF-α production is presented in ng/mL on the secondary Y axis (right hand). *Lactobacillus plantarum* WCFS-1, *E. coli* K12, BCG and LPS at 0.2 μg/mL were included as controls

**Fig. 5:**
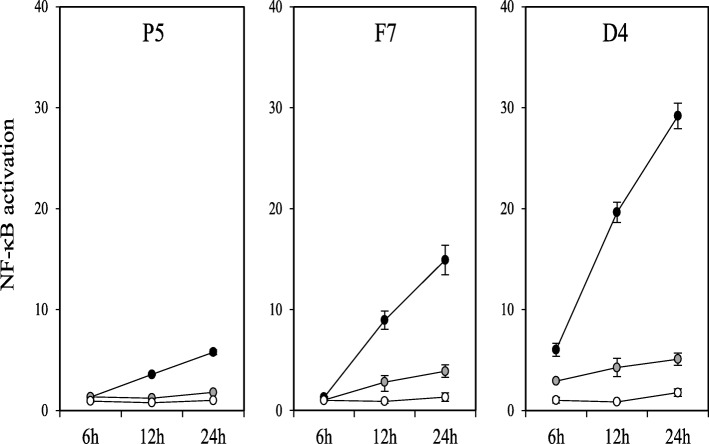
NF-κB activation in PMA-differentiated THP-1 macrophages exposed to inactivated cells of *Lactobacillus plantarum* P5, *Pediococcus lolii* F7 and *Lactobacillus reuterii *D4 at a bacteria:macrophage ratio of 100:1 (black dots); 10:1 (grey dots); or 1:1 (white dots) for 24 h. NF-κB activation is presented as a fold increase over a non-stimulated condition and the selected LAB strains represent a low (P5), moderate (F7), and high (B4) NF-κB response in THP-1 macrophages. Data represent at least two biological replicates

To study the effect of LAB on NF-κB activation we employed THP-1 macrophages carrying a NF-κB luciferase reporter as a model system. Cells were differentiated with phorbol 12-myristate 13-acetate (PMA) for 48 h and subsequently exposed to the 12 LAB isolates. We assessed NF-κB responses at 100 bacteria per macrophage, a dose that had been previously observed to trigger activation by LAB (data not shown). We found that exposure to inactivated weissella and enterococci isolates did not trigger NF-κB luciferase activation. However, a significant increase in the reporter activity was recorded upon exposure to pediococci and lactobacilli isolates (see white bars in Figure 4). Interestingly, activation was strain-specific. Isolates *P. pentosaceus* B4, *P. acidilactici* I32 and *L. plantarum* P5 showed a low NF-κB activation that is directly comparable to that observed with the human probiotic *L. plantarum* WCFS-1, with no significant differences between them. By contrast, isolates *P. acidilactici* E24, *E. faecalis* F7 and *P. acidilactici* M16 displayed a significantly higher pro-inflammatory effect by comparison with WCFS-1 (*p* < 0.05), with a moderate NF-κB luciferase activation as observed for LPS at 0.2 μg/mL. Interestingly, isolate *L. reuterii* D4 induced a NF-κB response that was as high as that observed with other bacteria associated with a high pro-inflammatory profile such as *E. coli* and a BCG Pasteur strain, with no significant differences between them. We then assessed activation of the NF-κB-driven luciferase at different time points (6, 12 and 24 h post-exposure) and doses (100, 10 and 1 bacteria per macrophage) with three representative LAB (low, medium, and high NF-κB induction) (Figure 5). In all cases, LAB activated NF-κB in a dose-dependent manner.

### Production of TNF-α in macrophages exposed to LAB

To confirm that the NF-κB activation observed by the luciferase reporter led to a production of an inflammatory cytokine, the levels of tumour necrosis factor alpha (TNF-α) were measured in the medium of macrophages exposed to LAB. TNF-α is a critical NF-κB-dependent cytokine commonly found as a host inflammatory marker in response to bacteria [22]. PMA-differentiated THP-1 cells were exposed to 100 bacteria per macrophage for 24 h and supernatants were tested in an anti-TNF-α ELISA. TNF-α production correlated positively with NF-κB activation (Additional file 1: Figure S5). Enterococci and weissella were unable to induce any TNF-α production, whereas pediococci and lactobacilli mediated variable amounts of TNF-α production (black bars in Figure 4). These results confirmed not only that the pro-inflammatory role of our LAB isolates is dependent on the species but also that isolates of pediococci and lactobacilli induce variable pro-inflammatory levels in a strain-specific manner.

### Influence of LAB on BCG-induced NF-κB activation

**Fig. 6:**
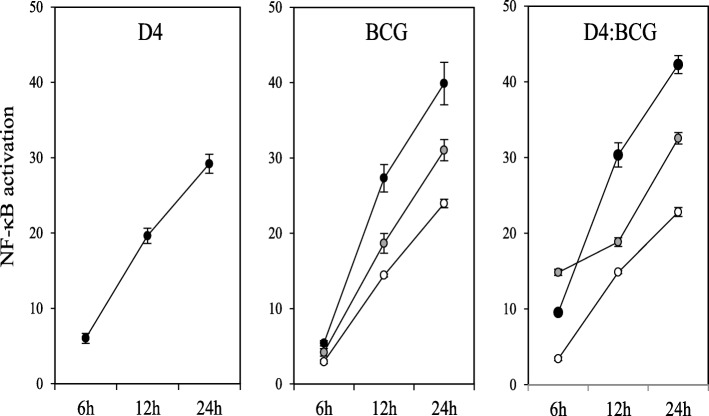
NF-κB activation in PMA-differentiated THP-1 macrophages exposed to inactivated cells of *Lactobacillus reuterii* D4, BCG and combinations of D4 with BCG (D4:BCG) for 24 h. When the bacteria were tested on their own, macrophages were exposed to bacteria at a bacteria:macrophage ratio of 100:1 (black dots); 10:1 (grey dots); and 1:1 (white dots); for the D4:BCG combinations, the macrophages were challenged with bacteria at a LAB:BCG:macrophage ratio of 100:100:1 (back dots); 100:10:1 (grey dots); and 100:1:1 (white dots). NF-κB activation is presented as a fold increase over a non-stimulated condition and data represent at least two biological replicates

**Fig. 7:**
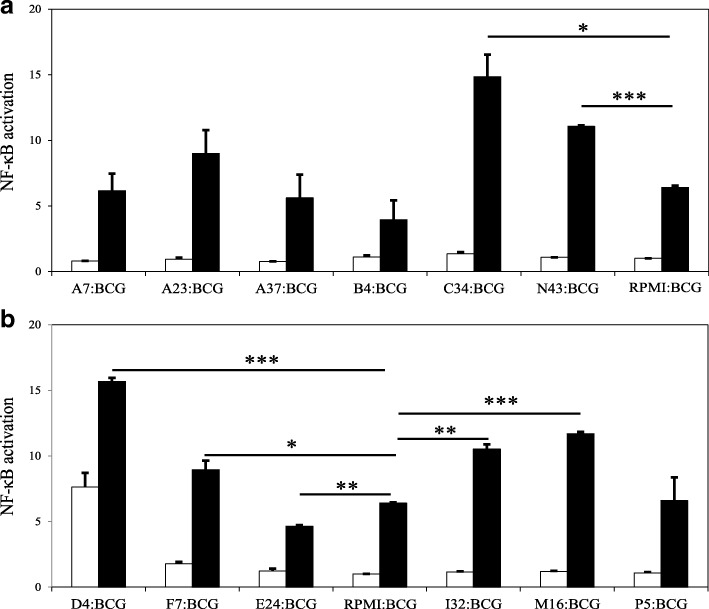
NF-κB activation in PMA-differentiated THP-1 macrophages exposed to inactivated cells from badger LAB isolates with no (or very low) pro-inflammatory (a) or pro-inflammatory (b) profile, in the absence (white bars) or presence (black bars) of BCG at a ratio of 100:10:1 (LAB:BCG:macrophage) for 6 h. A medium-only condition (RPMI) was included as a control. Comparative statistical analysis was carried out between BCG and all the LAB-BCG combinations using the Student t-test (*p < 0.05, **p < 0.01, ***p < 0.005). Data are mean ± SD representing three biological replicates each

In order to test whether LAB isolates had a significant effect on the response of macrophages to BCG, we monitored NF-κB changes in macrophages exposed to a BCG Pasteur strain in combination with each of our LAB isolates. BCG is a potent activator of NF-κB and exposure to PMA-differentiated THP-1 cells for as little as 6 h is sufficient to detect significant changes in NF-κB reporter activity, either alone or in combination with LAB, especially at a ratio of 10 LAB per 1 BCG (Figure 6). Thus, we studied the effect of LAB on BCG-mediated NF-κB activation at a ratio of 100:10:1 (LAB:BCG:macrophage) after 6 h of co-incubation. In order to facilitate data interpretation, the results obtained from the macrophages exposed to the LAB-BCG combinations were divided into two groups based on the intensity of the pro-inflammatory profile of the LAB isolates on their own (Figure 7). Most LAB isolates with no, or very little, effect on NF-κB activation had a negligible impact on BCG-induced reporter activity (Figure 7a). However, isolates *E. faecalis* C34 and *W. paramesenteroides* N43, which had no effect on the NF-κB response in isolation for even 24 h (Figure 4), significantly increased NF-κB activation when combined with BCG. On the other hand, nearly all the isolates that clearly activated the NF-κB pathway on their own significantly enhanced the pro-inflammatory response of macrophages when combined with BCG (Fig. 7b). The exceptions were isolates *P. acidilactici* E24 and *L. plantarum* P5. Both P5 and E24 increased NF-κB activation when incubated in isolation with macrophages for 24 h (Fig. 4), but this was not observed after 6 h of co-incubation with BCG. The BCG-induced response remained unaffected by P5, but was surprisingly reduced by E24 and this was statistically significant.

## Discussion

This is the first publication reporting the isolation and identification of LAB from faeces of badgers. Previous works have reported only a single LAB isolate from the intestine of a badger [23] or a LAB population detected in faeces of other mustelids [24]. In our study we show that different LAB species isolated from different badgers can exert in vitro inhibitory activity against *M. smegmatis* and *M. bovis* BCG. The effect of the LAB isolates seems to be species-dependent. The weissella isolates only reduced BCG viability as measured by GFP expression, but had no significant effect on the survival rate of BCG as measured by bacterial counts, suggesting a more bacteriostatic, rather than bacteriocidal, effect. In contrast, isolates of enterococci, lactobacilli and pediococci displayed a bactericidal effect since they were capable of reducing significantly both the viability and survival rate of BCG.

We suspected that the activity that our LAB isolates displayed against *M. smegmatis* were due to the combination of acidic pH and metabolites such as lactic acid [25]. However, low pH and accumulation of organic acids, either alone or in combination, were insufficient to induce a decrease in the viability and survival of *M. bovis* BCG when grown as a monoculture or in acidic cell-free supernatants from LAB. Our finding is in agreement with a previous study that reported that *M. bovis* tolerates low pH and organic acids; being able to survive and remain virulent in very acidic environments such as cheese [26]. Interestingly, the 12 LAB isolates selected in our study caused a significant reduction in the viability and the survival of BCG when cultured together in vitro. These results are consistent with two previous studies that reported a reduction in *M. bovis* counts in co-cultures with LAB in milk [27, 28]. We also observed that this significant BCG reduction only took place when BCG and LAB were present in cultures together, suggesting the direct inhibition of BCG by LAB, rather than an indirect effect due to acidic conditions or the accumulation of toxic metabolites derived from LAB. Whether this inhibition is due to antimicrobial metabolites that are only produced in the presence of BCG is a question that remains to be elucidated. However, other studies have reported that the efficacy of LAB metabolites against bacterial pathogens is more evident when competing simultaneously in the same environment [29]. The relevance of our findings depends on the extent to which opportunities exist in vivo for resident LAB and orally administered BCG vaccine to come into intimate contact with one another. This is currently unknown and would need to be tested using more complex model systems, such as in vitro organ culture models. Nonetheless, LAB may still contribute to a gastric environment that is not conducive to BCG survival.

The reasons why our LAB isolates differ in their activity against BCG may lie in the differing metabolic profiles of the isolates. Weissella are obligate heterofermenters, able to produce not only lactic acid as the major end product from energy metabolism but also other metabolites such as acetic acid and ethanol [30]. The remainder of the isolates produce lactic acid as the main end product since they are obligate or facultative heterofermenters [31]. The amount of lactic acid produced by the isolates in vitro may change depending on the oxygen availability, but this organic acid seems to have a key role as the main antimicrobial metabolite. Besides, strains of pediococci, enterococci and lactobacilli have been reported to produce antimicrobial peptides such as pediocins [32], enterocins [33] and plantaricins [34], as well as reuterin [35], which could act synergistically with lactic acid against BCG.

Oral administration of probiotics has been suggested as a convenient and safe way to improve the efficacy of conventional vaccines within communities [36]. Surface and/or secreted proteins and metabolites of LAB act as inflammatory mediators, interacting with cells of the mammalian immune system to stimulate and modify immunity [37–39], typified by activation of the NF-κB pathway in macrophages. Whilst studies are relatively limited in the context of oral vaccination, it would appear that efficient activation of the NF-κB pathway following oral immunisation results in strong systemic immunity [40]. Much of the research on the role of probiotics in the immune response has been conducted with lactobacilli, but other studies using other LAB that are in close contact with the mucus layer and epithelial crypts of the small intestine such as enterococci and pediococci, have also demonstrated an immune modulation that enhanced vaccine efficacy [41–43]. Therefore, we wanted to determine the impact that different badger LAB isolates had on the inflammatory response, on their own, and in the context of BCG vaccination.

In this study we have observed that inactivated cells of pediococci and lactobacilli induce NF-κB activation and TNF-α production in the human THP-1 macrophage cell line. On the contrary, inactivated cells of weissella and enterococci had no effect either on the NF-κB pathway or on TNF-α production. As previously reported by Jensen et al. [38], our *Lactobacillus reuterin* isolate induced the highest pro-inflammatory response, with TNF-α production, whereas *L. plantarum* was a much lower inducer, as observed with the human probiotic *L. plantarum* WCFS-1. When compared to *E. coli* and BCG, the pediococci isolates induced a moderate pro-inflammatory response in THP-1 macrophages. This response was observed to be strain-dependent as some of the isolates were much higher inducers than others. More detailed studies are needed to fully understand the pro-inflammatory effect of pediococci but a recent study has reported that exopolysaccharides from a *Pediococcus pentosaceus* strain induce the production of pro-inflammatory cytokines via NF-κB [44]. Furthermore, our experiments with inactivated cells of enterococci did not show any NF-κB activation although heat-killed enterococci can activate the NF-κB pathway in murine macrophages [39]. This difference could be explained by the fact that our bacteria and macrophages derive from strains and species that are different to the previous work.

Combinations of BCG with inactivated cells from almost all our lactobacilli and pediococci resulted in enhanced NF-κB activation, or at least, in a non-detrimental effect on this pro-inflammatory pathway. On the basis of other work [45], it is possible this is effect is mediated through TLR-2. The only exception was *P. acidilactici* E24, which induced NF-κB activation in isolation, but reduced the extent of activation triggered by BCG. Takata et al. reported that *P. acidilactici* inoculation in mice suppressed the production of inflammatory cytokines, whilst increasing the number of interleukin (IL)-10-producing T cells [46]. It is well-known that IL-10 and other anti-inflammatory cytokines have the capacity to modulate NF-κB activation [47]. Further investigation is required to determine whether the effects observed for *P. acidilactici* in mice also occur in badgers.

Although inactivated cells of weissella and enterococci were unable to cause any pro-inflammatory activation in THP-1 macrophages, co-cultures of certain strains with BCG resulted in a synergistic enhancement of NF-κB responses. Previous studies have reported that heat-killed weissella and *E. faecalis* proteins block the secretion of pro-inflammatory cytokines and attenuate NF-κB activation [48, 49], but also that lipoteichoic acids of weissella and enterococci significantly increase the secretion of pro-inflammatory cytokines and contribute to host inflammatory responses [48, 50]. Therefore, there could be a significant variability between weissella and enterococci strains, with some of them able to modulate the outcome of BCG-induced inflammatory responses; possibly via TLR2, as both LAB and BCG activate macrophages in a TLR-2-dependent manner [37, 45].

Despite the fact that we did not use monocytes from badgers, our model has proved to be informative; generating preliminary data that show the potential for badger-derived LAB to cause immunomodulation, either alone or in combination with BCG. This now needs to be tested using macrophages isolated from badgers. In addition, although the mechanisms that our isolates utilize as immunomodulators remain to be elucidated, our data also raise the interesting possibility of their potential exploitation as adjuvants to BCG if used as inactivated cells.

## Conclusions

We started from the premise that the variation and activity of commensal gut bacteria could play an important, and hitherto underappreciated role in the outcome of oral BCG vaccination in badgers. We found that LAB isolated from badger faeces could influence the viability of BCG vaccine in vitro and modify the pro-inflammatory response using an established macrophage-like model. These preliminary results, although generated from reductionist in vitro assays, suggest our premise is worthy of further investigation. More extensive evaluation of the badger gut microbiome and its impact on the efficacy of oral vaccination in vivo appears warranted.

## Methods

### Badgers and sampling

Faecal samples were collected from a total of 26 badgers kept in captivity at the Animal Plant and Health Agency for other scientific projects authorised under a UK Home Office Project Licence and Ethical approval by the Agency. These badgers were initially taken from a bTB-free wild population and they all tested negative to *M. bovis* infection following a quarantine procedure that consisted of bTB testing on three sequential occasions. The TB testing was carried out at approximately one month intervals using the badger interferon (IFN)-γ ELISPOT, the badger specific IFN-γ release assay (IGRA) and a standard *M. bovis* culture with clinical samples. Three negative tests were required before an animal was considered free of bTB and admissible to the study. All the badgers were on a diet that mainly consisted of commercial dog food with no probiotics, a very high source of carbohydrates from wholegrain maize and wheat (58%), a high source of protein from fish (20%), a low level of fat (7%) -most of it as polyunsaturated fatty acids; and enriched with fibre (4%) and essential vitamins and minerals. The badgers were also provided with commercial dry dog food (no LAB in its composition), peanuts, apples and sweet chestnuts, when available, as well as any food from foraging activities such as insects, worms and mice. Fresh water was available ad libitum*.* Faecal samples were taken from badgers using rectal swabs following intramuscular administration of ketamine hydrochloride (10 mg/kg, Vetalar, Boehringer Ingelheim), medetomidine hydrochloride (0.1 mg/kg, Domitor, Pfizer), and butorphanol (0.1 mg/kg, Torbugesic R, Zoetis UK Ltd) as described previously [11]. Badgers were then weighed, examined for any signs of disease or injury and their corresponding sex noted to ascertain whether these factors could affect the different levels of LAB found in the collected faecal samples.

### Isolation of LAB from faecal samples

The rectal swabs were immersed in maximum recovery diluent (Oxoid) to prepare serial dilutions that were spread onto MRS agar plates (Oxoid) for the isolation of LAB. The serial dilutions were also spread onto PCA plates (Oxoid) to estimate the percentage of LAB when compared to total microaerophilic viable bacteria. Plates were incubated at 37 °C for 48–60 h under microaerophilic conditions. A total of 50 different colonies were then selected from MRS-agar plates containing approximately 20–100 colonies from each of the 26 collected faecal samples (a total of 1300 colonies), and cultivated in Thermo Scientific Nunc MicroWell™ 96-well plates with MRS broth at 37 °C for 48–60 h under microaerophilic conditions.

### Antimycobacterial screening of LAB

The supernatants of the MRS cultures present in the 96-well plates were tested against *Mycobacterium smegmatis* mc^2^ 155 [51] using the spot-on-agar test. Briefly, the 96-well plates were centrifuged for 10 min at 5000 rpm to deposit 5 μL of the culture supernatants on TSA plates which had been swabbed with *M. smegmatis*. The MRS cultures whose supernatants showed antimicrobial activity against *M. smegmatis* were selected to confirm their antimycobacterial activity using the Agar-well Diffusion Test (ADT) [52]. The nisin-producer *Lactococcus lactis* NZ9700 and its corresponding knock-out strain NZ9800 were used as positive and negative controls, respectively [53].

### Bacterial strains and growth conditions

All LAB, including the badger isolates and the human probiotic *L. plantarum* WCFS-1 [54], were grown in MRS broth/agar (Oxoid) at 37 °C without any aeration for 24 h. The *L. lactis* strains NZ9700 and NZ9800 [55] were also grown static in MRS for 24 h but at 30 °C. The strains *E. coli* ATCC25922, *E. coli* K12 [56] and *S. enterica* ATCC14028 were propagated in Trypticase Soy Broth/Agar (TSB/TSA, Oxoid) at 37 °C for 24 h. *M. smegmatis* mc^2^ 155 was also cultured in TSB at 37 °C but supplemented with 0.05% Tween 80 (Sigma Aldrich) and for 48 h. The two *M. bovis* strains BCG Δ*leuD* pAS^OriM^XF [57] and BCG Pasteur [58] were cultured in Middlebrook 7H9 (Difco) supplemented with 10% Oleic acid albumin dextrose-catalase (OADC) enrichment (BD BBL™), 0.1% Tween 80 (Sigma Aldrich) and 0.2% glycerol (Sigma Aldrich) or Middlebrook 7H11/7H10 (Difco) medium supplemented with 5% OADC and glycerol (2 mL/L) at 37 °C. BCG Δ*leuD* pAS^OriM^XF was previously generated by transforming BCG Pasteur Δ*leuD* with the mycobacterial episomal vector pAS^OriM^XF that complements the leucine mutation to correct auxotrophy and enable stable expression of GFP under the control of the constitutively expressed promoter pL5X [59]. All enterobacteria and mycobacteria were grown with aeration in an orbital shaker at 225 rpm and all bacterial strains were maintained as − 80 °C frozen stocks in their appropriate media with the addition of 15% glycerol.

### Identification and selection of LAB with antimycobacterial activity

Colonies from MRS cultures whose anti-mycobacterial activity was confirmed by ADT were identified based on their morphology (small, circular, white/creamy colonies), gram staining (positive) and oxidase/catalase tests (negative). Further identification was carried out by 16S rRNA gene sequencing (LGC Genomics) before a final selection of the 12 most representative LAB species comprising different bacterial species isolated from different animals but also different strains representing the most predominant bacterial species found.

### Co-cultures of BCG and LAB

Cell pellets of the selected LAB and the leucine auxotrophic strain BCG Δ*leuD* pAS^OriM^XF were obtained from cultures at their exponential phase of growth. The auxotrophy correction allowed us to perform co-cultures of LAB and BCG without using antibiotics while maintaining the GFP plasmid to monitor BCG viability over time. In order to prepare the co-cultures, 12 BCG pellets were resuspended with each LAB pellet using Mueller-Hinton (MH) broth supplemented with 10% Oleic acid-Albumin-Dextrose-Catalase (OADC) enrichment, 0.1% Tween 80 and 0.2% glycerol, to generate 12 co-cultures at an initial concentration of 5 × 10^3^ CFU/mL for BCG and 5 × 10^5^ CFU/mL for the LAB. Our own preliminary studies showed that MH broth supplemented with OADC, tween and glycerol is the optimal to support the growth of LAB without altering the viability and survival rate of BCG for 48 h when grown as mono-cultures. The co-cultures were then grown in an orbital shaker at 37 °C for 48 h and samples were collected at 0, 24 and 48 h post-incubation to determine BCG viability and survival rate. Co-cultures of BCG with the enterobacteria *E. coli* (ATCC 25922) and *S. enterica* serovar Typhimurium (ATCC 14028) at an initial concentration of 5 × 10^5^ CFU/mL were also included in the study for comparative purposes.

### Viability and survival rate experiments

In order to monitor BCG viability in the co-cultures, aliquots of 100 μl were transferred into Thermo Scientific Nunc MicroWell™ 96-well plates to measure fluorescence emission at 485/535 nm in a DTX 880 Multimode Detector microplate reader (Beckman Coulter). Simultaneously, BCG survival rate was determined by total bacterial counts on BCG-selective agar plates (Middlebrook 7H10 supplemented with 5% OADC and 0.2% glycerol). Bacterial counts were also carried out for LAB and the enterobacteria on their corresponding selective agar plates: MRS and Violet Red Bile Glucose (Oxoid), respectively. BCG viability was expressed as log_10_ RFU (Relative Fluorescence Units), where measured fluorescence units (FU) were multiplied by the log_10_ increase in the CFU/mL of the competitor and normalized with the highest increase in CFU/mL observed for any of the competitors. These relative units let us compare GFP expression between the co-cultures at each time point over 48 h since the bacterial counts varied amongst the LAB and enterobacteria (Additional file 1: Figure S6). The BCG survival rate was presented as CFU/mL.

### pH in co-cultures

The pH of all co-cultures was measured using a Hanna pH meter at time points 0, 24 and 48 h, starting from an initial pH of 7 for the media, to determine the effect of pH on the viability and survival of BCG while co-cultured with LAB. The viability and survival rate of BCG was also monitored at pH 7 and pH 5 as explained above but as a monoculture to evaluate the influence of pH on the growth of BCG.

### Metabolite accumulation experiments

In order to determine the effect of LAB metabolites on BCG growth, cell pellets of BCG Δ*leuD* pAS^OriM^XF were resuspended in cell-free supernatants collected from 48 h cultures of LAB, *E. coli* ATCC25922 and *S. enterica* ATCC14028 to monitor viability, and survival rate as explained above. The BCG survival rate was indicated as CFU/mL and the viability as log_10_ FU (Fluorescence Units).

### Culture and differentiation of THP-1 cells

The THP-1 Lucia™ NF-κB monocyte cell line (Invivogen) was used to monitor the NF-κB signal transduction pathway in macrophages when exposed to inactivated cells obtained from the badger LAB isolates. These macrophages secrete Lucia luciferase under the control of the NF-κB promoter. The cells were grown in Roswell Park Memorial Institute (RPMI) 1640 (Life Technologies) supplemented with 15% foetal bovine serum (FCS, Seralab) and 1% Penicillin/Streptomycin (Pen/Strep, Life Technologies) at 37 °C in an atmosphere of 5% CO_2_. PMA (Santa Cruz Biotechnology) was dissolved in DMSO at 10 mg/mL. 5 × 10^4^ cells per well were seeded in with RPMI supplemented with 20 ng/mL PMA in Thermo Scientific Nunc MicroWell™ 96-well plates. After 48 h, medium was replaced with RPMI containing 2% FCS and 1% Pen/Strep supplemented with inactivated cells of LAB.

### Luciferase measurements from THP-1 cells exposed to LAB

PMA-differentiated THP-1 cells were exposed to heat-inactivated (70 °C, 2 h) LAB pellets that were resuspended in RPMI containing 2% FCS and 1% Pen/Strep. At the indicated times, supernatants were transferred to white-bottom 96-well plates and luciferase activity was measured in the presence of 2 μg/mL of coelenterazine (NanoLigh Technology) using a Clariostar plate reader (BMG Biotech). The BCG Pasteur strain, *E. coli* K12 and *L. plantarum* WCFS1, all as inactivated cells, were used as controls, as well as LPS at 0.2 μg/μl (Sigma Aldrich). The BCG Pasteur strain was also used to test whether LAB isolates had a significant effect on the response of macrophages to BCG. NF-κB activation was calculated as a fold increase over the measurements recorded for unchallenged macrophages.

### ELISA for TNF-α detection

TNF-α production was detected and quantified in the supernatants of PMA-differentiated THP-1 cells using the eBioscience Human TNF-α ELISA Ready-SET-Go kit as indicated by the manufacturer’s instructions.

### Statistical analysis

Statistical analysis was performed using GraphPad Prism version 7.00 for Windows (GraphPad Software, La Jolla California USA, www.graphpad.com)**.** Data are mean ± SD, representing three biological replicates, unless indicated. Differences between time-points for the same samples were analysed by the Student *t*-test, while differences between samples at different time points were analyzed by one way ANOVA followed by Fisher’s Least Significant Difference (LSD) Test.

## Additional file

Additional file 1: **Figure S1.** pH of BCG co-cultures with LAB isolates or *E. coli* and *Salmonella* after 0 (white bars), 24 (grey bars) and 48 (black bars) h of incubation. Different letters signify statistical differences between mean values using ANOVA followed by LSD (*p* < 0.05).
**Figure S2.** Viability (A) and survival rate (B) of BCG when grown as a monoculture at pH 5 or pH 7 after 0 (white bars), 24 (grey bars) and 48 (black bars) h of incubation. Viability and survival rate are expressed as log_10_ Fluorescence Units (FU) and log_10_ CFU/mL, respectively.
**Figure S3.** BCG viability when grown in cell-free supernatants from 48 h-cultures of enterococci (i), weissella (ii), lactobacilli (iii), pediococci (iv) and gut-associated Gram-negative bacteria (v) after 0 (white bars), 24 (grey bars) and 48 (black bars) h of incubation. BCG viability is expressed as log_10_ Fluorescence Units (FU).
**Figure S4.** Survival rate of BCG when grown in cell-free supernatants from 48 h-cultures of enterococci (i), weissella (ii), lactobacilli (iii), pediococci (iv) and gut-associated Gram-negative bacteria (v) after 0 (white bars), 24 (grey bars) and 48 (black bars) h of incubation. BCG survival rate is expressed as log_10_ CFU/mL.**Figure S5.** Significant positive correlation between mean values of NF-κB activation and TNFα production in PMA-differentiated THP-1 macrophages exposed to inactivated bacterial strains and LPS, at a ratio of 1:100 (macrophage::bacteria) and 0.2 μg/μl, respectively (*p* < 0.0001).
**Figure S6.** Growth rate of enterococci (i), weissella (ii), lactobacilli (iii), pediococci (iv) and gut-associated Gram-negative bacteria (v) after 0 (white bars), 24 (grey bars) and 48 (black bars) h of incubation when co-cultured with BCG. Growth rate is expressed as log_10_ CFU/mL. (DOCX 217 kb)

## Abbreviations

BCG: Bacillus Calmette-Guerin
bTB: Bovine tuberculosis
LAB: Lactic acid bacteria
