# Supplementary material for: Lactic acid Bacteria isolated from European badgers (Meles meles) reduce the viability and survival of Bacillus Calmette-Guerin (BCG) vaccine and influence the immune response to BCG in a human macrophage model
Source: BMC Microbiol. 2018 Jul 13;18:74. doi: 10.1186/s12866-018-1210-z (PMC6044090; doi:10.1186/s12866-018-1210-z)
Supplement: Supplementary file 1 — Figure S1. pH of BCG co-cultures with LAB isolates or E. coli and Salmonella after 0 (white bars), 24 (grey bars) and 48 (black bars) h of incubation. Different letters signify statistical differences between mean values using ANOVA followed by LSD (p < 0.05). Figure S2. Viability (A) and survival rate (B) of BCG when grown as a monoculture at pH 5 or pH 7 after 0 (white bars), 24 (grey bars) and 48 (black bars) h of incubation. Viability and survival rate are expressed as log10 Fluorescence Units (FU) and log10 CFU/mL, respectively. Figure S3. BCG viability when grown in cell-free supernatants from 48 h-cultures of enterococci (i), weissella (ii), lactobacilli (iii), pediococci (iv) and gut-associated Gram-negative bacteria (v) after 0 (white bars), 24 (grey bars) and 48 (black bars) h of incubation. BCG viability is expressed as log10 Fluorescence Units (FU). Figure S4. Survival rate of BCG when grown in cell-free supernatants from 48 h-cultures of enterococci (i), weissella (ii), lactobacilli (iii), pediococci (iv) and gut-associated Gram-negative bacteria (v) after 0 (white bars), 24 (grey bars) and 48 (black bars) h of incubation. BCG survival rate is expressed as log10 CFU/mL.Figure S5. Significant positive correlation between mean values of NF-κB activation and TNFα production in PMA-differentiated THP-1 macrophages exposed to inactivated bacterial strains and LPS, at a ratio of 1:100 (macrophage::bacteria) and 0.2 μg/μl, respectively (p < 0.0001). Figure S6. Growth rate of enterococci (i), weissella (ii), lactobacilli (iii), pediococci (iv) and gut-associated Gram-negative bacteria (v) after 0 (white bars), 24 (grey bars) and 48 (black bars) h of incubation when co-cultured with BCG. Growth rate is expressed as log10 CFU/mL. (DOCX 217 kb) [file 12866_2018_1210_MOESM1_ESM.docx]

**Fig. S1**

**Fig. S2**

**Fig. S3**

**Fig. S4**

**Fig. S5**

**Fig. S6**
